# Lightweight Ensemble Learning based on Post-Hoc Probability Fusion with Its Application to Lung CT Image Classification

**DOI:** 10.2174/0115734056474074260630195403

**Published:** 2026-07-09

**Authors:** Yuqian Feng, Yang Qu

**Affiliations:** 1 Department of Respiratory and Critical Care Medicine, Union Hospital, Tongji Medical College, Huazhong University of Science and Technology, Wuhan, 430030, China; 2 School of Naval Architecture and Ocean Engineering, Huazhong University of Science and Technology, Wuhan, 430074, China

**Keywords:** Lightweight ensemble learning, Weighted probability fusion, Model integration, Computed tomography imaging, Pulmonary disease classification, Lung CT Image

## Abstract

**Introduction:**

Deep learning model fusion has become one of the most effective methods to improve the performance of ensemble learning frameworks. However, the optimization of critical parameters within ensemble learning models to improve prediction accuracy remains a challenging issue. This study proposes a lightweight ensemble learning model fusion method based on post-hoc weighted averaging of predicted probabilities..

**Methods:**

By appropriately adjusting the fusion weight coefficient and the decision threshold, the optimal fusion strategy is investigated to improve performance metrics including accuracy, precision, recall, and F1 score. To verify the effectiveness of the proposed lightweight ensemble learning fusion method, it is applied to a medical classification task using lung computed tomography (CT) images, which aims to distinguish between pneumonia and lung cancer, two diseases with inter-class visual similarity. We construct a dataset containing 859 lung CT images representing pneumonia and lung cancer. First, we evaluated nine deep learning architectures from the VGG family (VGG11, VGG13, VGG16, VGG19) and the ResNet family (ResNet34, ResNet50, ResNeXt50, ResNet101, ResNeXt101). The results show that ResNet-based models consistently outperform VGG-based models. Therefore, we adopt the proposed lightweight weighted probability fusion strategy to integrate two complementary ResNet models, namely ResNet34 and ResNeXt50.

**Results:**

Experimental results show that the optimized fusion model achieves an accuracy of 96.67%, precision of 94.68%, recall of 98.88%, and F1 score of 96.74%, outperforming the individual models across all evaluation metrics.

**Discussion:**

These results show that the proposed lightweight ensemble learning method can enhance classification accuracy and decision robustness. This suggests that weighted probability fusion is effective for tasks with inter-class visual similarity.

**Conclusion:**

The proposed method provides a feasible strategy for a model ensemble framework in medical image analysis, particularly when balancing performance and computational efficiency.

## 1 INTRODUCTION

Enhancing the prediction accuracy, generalization capacity, and system robustness of machine learning models has emerged as a challenging research area. Nonetheless, individual machine learning models have their own limitations in expressive power and feature extraction capabilities, which significantly constrain their effectiveness in complex pattern recognition tasks. In contrast, ensemble learning techniques improve prediction accuracy, generalization, and robustness by integrating multiple base models to form multi-model ensemble systems. Despite the widespread development and application of various ensemble learning methods, most of them require substantial computational resources or involve intricate training procedures, thereby restricting usability in real-time or resource-limited cases. Consequently, the development of efficient and lightweight ensemble model fusion strategies is essential to achieve an optimal trade-off between model performance and computational efficiency. This study introduces a lightweight model fusion approach and applies it to the classification of lung computed tomography (CT) images characterized by high inter-class similarity. A series of comparative experiments verifies the effectiveness of the proposed lightweight ensemble learning method.

### 1.1 Ensemble Learning

Ensemble learning serves as a well-known machine learning architecture that effectively combines multiple base learners to improve prediction accuracy and has been effectively employed in different complex pattern recognition tasks. This approach combines data fusion, modeling, and data mining within a uniform framework, thereby addressing a broad spectrum of practical machine learning challenges [1]. By training diverse models and aggregating their outputs, ensemble learning enhances the generalization capability of the fusion model [2]. Existing review literature offers a thorough examination of the theoretical foundation of ensemble learning, detailing both traditional and novel ensemble methodologies while clarifying current research trends and prospective themes [2]. Importantly, ensemble methods integrate predictions from multiple base models, consistently achieving good performance across diverse application domains [3]. Additionally, these studies clearly clarify various ensemble learning strategies, with emphasis on three principal techniques: bagging, boosting, and stacking, and provide insights into their variants [3].

To enhance the prediction capabilities of ensemble learning comprehensively, many ensemble strategies have been developed, resulting in the establishment of numerous sophisticated ensemble fusion architectures. Presently, ensemble learning techniques mainly encompass bagging, boosting, and stacking, each demonstrating outstanding performance improvements across diverse application domains [3]. Ensemble learning mitigates the prediction constraints inherent in individual models by integrating the outputs of multiple base learners. Among these approaches, weighted ensemble fusion methods assign specific weights to each base learner, thereby accounting for their prediction strengths across different categories [4]. Additionally, the integration of deep neural networks (DNNs) with swarm intelligence provides an emerging research area within ensemble learning. These approaches employ DNNs as base learners and incorporate swarm-based decision-making mechanisms to facilitate effective model fusion [5]. Moreover, random forests, a classical ensemble technique, have achieved substantial advancements by constructing multiple uncorrelated decision trees and aggregating their predictions, leading to prominent accuracy improvements in various classification tasks [2]. Consequently, ensemble learning methods capitalize on the complementary strengths of multiple base learners, effectively addressing the limitations of single models and enhancing prediction performance [6].

Ensemble learning offers outstanding performance enhancements compared to individual models. In the field of medical image analysis, which depends heavily on specialized and standardized imaging data, ensemble methods present a particularly promising avenue for application. The construction of a typical ensemble model generally involves two primary stages: the generation of base learners and the integration of these learners into an ensemble framework [5]. At present, ensemble learning techniques have been widely adopted in medical applications. Further development of deep learning has facilitated access to large-scale public medical image datasets. Notably, convolutional neural networks (CNNs) are widely used in tasks such as lung nodule analysis and cancer detection [7]. The majority of artificial intelligence applications in pulmonary imaging concentrate on X-ray and CT images. Deep learning systems have achieved, and in some cases surpassed, the diagnostic performance of human radiologists [7]. Consequently, ensemble learning strategies are widely implemented in medical image analysis, as these approaches integrate multiple models or aggregate diverse prediction outcomes to enhance both the accuracy and robustness of diagnostic predictions [8].

### 1.2 Lung CT Image Classification

Lung cancer is one of the most deadliest cancers worldwide, causing more deaths annually than breast cancer, prostate cancer, and colorectal cancer combined. The overall five-year survival rate for lung cancer remains below 20%, primarily due to its frequent presentation with clinical symptoms at advanced stages, which contributes to its high mortality rate [9, 10]. In contrast, pneumonia is a common acute pulmonary condition that may result from exposure to chemical or physical irritants, immune system impairment, infectious agents, or inappropriate medication use. The majority of pneumonia cases respond favorably to prompt diagnosis and appropriate treatment [11]. CT is widely used as an imaging modality for the detection of lung lesions. However, pneumonia and lung cancer can exhibit overlapping radiographic features on chest CT scans, including pulmonary consolidation, ground-glass opacities, and nodular shadows. This similarity poses a diagnostic challenge for radiologists, increasing the risk of misclassification between benign and malignant lesions, which can affect clinical decision-making and patient outcomes [7, 12].

Artificial intelligence (AI) is a broad term without a single, universally agreed-upon definition. It typically refers to computational methods that can learn from data and carry out specific tasks. As a subset of AI, deep learning has attracted considerable interest because it does not require explicit programming and has been rapidly developed in medical imaging applications [13]. For example, convolutional neural networks are widely used to detect lung nodules and lung cancer. Unlike traditional machine learning approaches that depend on manual feature extraction, deep learning directly handles raw image data, automatically extracts features, and quickly performs pattern recognition [7]. Early deep learning applications mainly handle lung nodule detection in X-ray and CT scans. Development in computer hardware and software has equipped modern systems with strong graphics processing capabilities. Additionally, the easy availability of large image datasets has facilitated the wide implementation of deep learning in medical imaging scenarios [14, 15]. Based on deep learning’s robust feature extraction and pattern recognition abilities, it has shown great success in detecting and classifying lung diseases, with accuracy levels approaching those of radiologists [16, 17].

Currently, deep learning models like VGG and ResNet have been effectively used for clinical analysis of lung CT scans [11, 18]. While numerous deep learning studies have investigated lung disease detection and classification, some of them have concentrated on a single imaging modality or a single model, with only a few comparing and evaluating multiple deep learning models using the same dataset [19]. Because CT images of various lung diseases often share similar radiographic characteristics, relying on a single model could affect its effectiveness and key metrics such as prediction accuracy. Additionally, since different models can produce conflicting classification outcomes for the same CT image, ensemble learning approaches that use their complementary strengths present a promising strategy to enhance classification performance [20, 21].

### 1.3 Main Work and Contributions

To handle the optimization-challenging issues of ensemble model fusion, this study introduces a lightweight ensemble fusion approach based on post-hoc weighted averaging of predicted probabilities. This technique will calibrate two critical parameters, namely fusion weight coefficients and decision thresholds, to derive an optimal fusion strategy, thereby enhancing performance metrics including accuracy, precision, recall, and F1 score. The proposed lightweight ensemble fusion method was evaluated *via* a CT medical image classification task. A dataset comprising 859 lung CT images, exclusively representing lung cancer and pneumonia cases, was collected and partitioned into 503 training samples, 180 testing samples, and 176 validation samples. Additionally, the study investigated the prediction performance of nine deep neural network architectures in binary classification tasks, including the VGG series (VGG11, VGG13, VGG16, VGG19) and the ResNet series (ResNet34, ResNet50, ResNeXt50, ResNet101, ResNeXt101). Experimental results show that ResNet architectures outperform VGG models, owing to their residual learning framework. Based on this insight, this paper proposes a lightweight ensemble fusion method that integrates paired ResNet models. This approach involves computing the prediction probabilities for each model, performing a post-hoc weighted average of these probabilities, and subsequently applying a threshold to determine the final classification result. Consequently, the weighted fusion of two models mitigates the prediction errors of individual models, thereby enhancing the classification performance of the whole ensemble framework. The main contributions of this paper are summarized as follows:

(1) Compared to the existing ensemble techniques such as Bagging and Boosting, this paper introduces a lightweight ensemble model fusion approach. Utilizing high-performance pretrained models of various ResNet architectures, the proposed ensemble method developed a weighted averaging scheme of the prediction probabilities generated by each base model. It does not need to retrain these base models while effectively utilizing their complementary advantages.

(2) Unlike some approaches that depend on opaque meta-learners, such as stacking algorithms, this study presents a lightweight ensemble fusion method characterized by interpretable parameters with clear physical meaning, *i.e*., fusion weight coefficients and decision thresholds. The optimization of these parameters through calibration enhances multiple performance metrics of the ensemble fusion model, such as accuracy and F1 score. This methodology circumvents complex hyperparameter tuning processes, thereby keeping the model simple.

(3) The proposed lightweight ensemble learning framework establishes a standardized benchmark for the comparative assessment of deep learning models and promotes the advancement of ensemble strategies in the field of medical image analysis. This paper delivers a precise computer-aided diagnostic tool for clinical application and offers researchers in related fields a reliable baseline model for further investigation.

## 2 RELATED WORK

As mentioned earlier, ensemble learning can enhance the performance metrics of fusion models. This section aims to present the theoretical foundations, operational mechanisms, and prevalent techniques of ensemble learning, with particular emphasis on the primary challenges encountered in practical implementations.

### 2.1 Overview of Ensemble Learning

#### 2.1.1 Theoretical Foundations of Ensemble Learning

Ensemble learning derives its decisions from the aggregated predictions of multiple base learners. Instead of relying on a single model, this approach capitalizes on the complementary strengths of diverse models to enhance both prediction accuracy and generalization capabilities, thereby handling the inherent limitations and uncertainties associated with individual models [3]. Empirical evidence demonstrates that single models often encounter issues such as high variance, substantial bias, and inadequate accuracy. Ensemble learning mitigates these challenges by using model combination techniques to effectively reduce output variance and bias. For instance, the bagging algorithm mainly reduces variance without a prominent increase in bias, whereas boosting algorithms focus mainly on bias reduction. Consequently, ensemble learning methods usually have superior overall performance compared to single models [3]. Ensemble techniques can be categorized into parallel and sequential ensembles. Parallel ensembles simultaneously train multiple base models and integrate their outputs, such as bagging and random forests [1]. Sequential ensembles, on the other hand, use an iterative training method wherein each successive model aims to correct the errors of its predecessor, as shown in boosting algorithms [2]. Additionally, within parallel ensembles, if all base models utilize the same ensemble algorithm, the ensemble strategy is homogeneous; otherwise, it is referred to as heterogeneous [1, 2].

#### 2.1.2 Mechanisms and Challenges of Ensemble Learning

The efficacy of ensemble learning is mainly influenced by the accuracy and diversity of its constituent base models [22]. Accuracy refers to a model’s ability to generalize effectively to previously unseen data, whereas diversity necessitates that base models generate distinct errors on novel data, thereby reflecting intrinsic disagreements among individual models. Nonetheless, since base models are typically trained on the same dataset, some degree of correlation among them is inevitable, which may diminish diversity. To address this challenge, various ensemble techniques have been developed to foster diversity. For instance, bagging enhances diversity by varying the training data samples, while boosting promotes diversity through adaptive adjustment of sample weights [23].

Numerous methodologies have been proposed and implemented to optimally combine base models. Compared to conventional voting schemes, ensemble selection methods mainly choose a subset of base models for combination. Based on the performance variability among different models, selecting complementary base learners can yield superior ensemble outcomes [24]. Ensemble selection approaches are generally categorized into static and dynamic methods. Static methods use ranking or optimization techniques to select a fixed subset of base models during the training phase [25]. Whereas dynamic methods determine the subset based on the characteristics of each individual input sample. To fully consider the benefits of both approaches, hybrid methods that integrate elements of static and dynamic selection have been widely investigated.

#### 2.1.3 Main Methodologies of Ensemble Learning

Among the various ensemble learning techniques, Bagging, Boosting, and Stacking represent three prominent methodologies.

Bagging, exemplified by algorithms such as Random Forest, employs a guided sampling strategy to generate diverse training subsets. This approach involves creating training datasets through random sampling. Subsequently, these samples are independently and simultaneously trained using weak or base learners, and their predictions are aggregated *via* voting or weighted averaging to mitigate variance. Bagging is particularly good for high-dimensional datasets since the presence of missing values tends to increase variance. Hence, this elevates the risk of overfitting and impairs the model’s ability to generalize effectively to unseen data.

Boosting, proposed in 1990 [26], includes widely used methods such as AdaBoost and XGBoost. Unlike Bagging, Boosting sequentially trains weak learners. Each successive model puts greater emphasis on instances misclassified by its predecessor. This iterative reweighting process enables the algorithm to identify critical parameters and enhance the ensemble model performance. By directing subsequent learners to focus on previously misclassified samples, Boosting effectively reduces bias. After a fixed number of iterations, the ensemble strategy combines the base learners through weighted fusion. Moreover, boosting can be integrated with various parameter optimization techniques, which further mitigate the high bias commonly observed in shallow decision trees and logistic regression models.

Stacking, proposed in 1992 [27], establishes a hierarchical ensemble framework that uses the outputs of multiple base models as input features for training a meta-model [28]. With the aid of a typical two-layer configuration, the first layer uses several base learners trained on the original dataset, while the second layer adopts the predictions of these base learners as features to train a meta-learner, forming the complete stacked model. More precisely, base models are fitted to the training data. Their predictions on a validation set and the corresponding true labels are used to establish a new training set for the meta-learner [29]. The meta-learner is often used as a linear classifier or linear regression model, which has been verified to be an effective strategy for optimally combining the outputs of base models.

#### 2.1.4 Ensemble Strategies in Deep Learning

Ensemble systems generally have two fundamental stages: the generation of diverse base learners and the effective aggregation of their outputs [5]. Beyond traditional methods such as Bagging, Boosting, and Stacking [3], many model fusion techniques have been proposed. Weighted fusion gives a computationally efficient and straightforward strategy, combining model predictions either by averaging output probabilities or through majority voting based on classification outcomes. For example, Nakata *et al*. adopted soft voting for liver tumor classification in ultrasound imaging [29]. Hence, the direct assignment of distinct weight values determines each model’s contribution, potentially constraining performance improvements, particularly in ensembles comprising heterogeneous components.

To handle the limitations of equal-weighted fusion, performance-based fusion methods assign weights according to the base learners’ performance on validation datasets. Zhou *et al*. introduced a group-based fusion approach using the 2-additive generalized Shapley arithmetic interval-valued intuitionistic fuzzy Choquet aggregation operator applied to the decision-maker attribute matrix for weight determination [5]. Similarly, Fabricius *et al*. proposed a group-based fusion technique that assigns differential weights to base learners based on their performance in specific discriminative tasks [4].

Dynamic weighting methods improve adaptability by adjusting the weights of base learners. This depends on the prediction uncertainty for each specific input instance. Zhang *et al*. developed a deep ensemble dynamic learning network for COVID-19 diagnosis, using a two-stage Bagging framework to facilitate continuous diagnostic decision-making [30]. The utilization of meta-learners to optimize the combined predictions of base learners has gained widespread recognition. For example, Guo *et al*. proposed a hybrid model that uses a stacked algorithm to integrate meta-learners such as logistic regression, random forest, and support vector machine, thereby fusing multiple classifiers for MASLD ultrasound image classification [31].

An emerging approach in model fusion involves the hybrid integration of CNN and Transformer architectures, capitalizing on their complementary strengths. Nanni *et al*. combined various CNN architectures with Transformer models, including Deit, Vision Transformer (ViT), Swin, and Coat, to propose an ensemble model integrating both CNN and Transformer components [32]. In a similar vein, Abulfaraj *et al*. developed an ensemble framework that integrates predictions from ViT, Enhanced MobileNetV2, and DenseNet201 models using a voting strategy [33].

In summary, ensemble learning develops a machine learning framework that integrates two or more base learners, such as regression models or neural networks, to enhance prediction performance. Fundamentally, ensemble methods combine the outputs of multiple individual models to yield predictions that surpass the accuracy attainable by any single model [1, 2].

### 2.2 Learning-Based Methods for Pulmonary Medical Image Analysis

Significant developments have been achieved in the application of deep learning and ensemble learning techniques in the field of medical image analysis, especially concerning pattern recognition and processing capabilities for the diagnosis of pulmonary diseases. Current research mainly concentrates on the detection and classification of various cases such as pneumonia, lung cancer, and other respiratory disorders [7].

#### 2.2.1 Pneumonia Detection and Diagnosis

Deep learning techniques have been widely used in the detection and diagnosis of pneumonia, leading to the development of various network architectures and strategies aimed at optimizing performance. Yang *et al*. [34] conducted an evaluation of five deep learning models over different scenarios to evaluate their pneumonia recognition capabilities. Through the identification of pneumonia patterns in chest X-rays and subsequent comparative analyses, they identified the most appropriate model for each specific context, thereby enhancing the efficiency of pneumonia detection. Wang *et al*. [35] introduced an attention-based classification approach based on the DenseNet architecture, combining a Squeeze-and-Excitation module to amplify pneumonia-related features within the feature maps, which resulted in improved classification accuracy. In a similar vein, Bundea *et al*. [36] proposed an attention-driven pneumonia classification method using three DenseNet variants—DenseNet121, DenseNet169, and DenseNet201. Trained on a dataset comprising 5,856 labeled X-ray images, their approach demonstrated enhanced diagnostic accuracy. Deepak *et al*. [37] developed a multi-stage diagnostic pipeline using multiple CNN models, namely SqueezeNet, ResNet50, and EfficientNet, in sequential detection phases, achieving fast pneumonia diagnosis with accuracy over 97%. Slama *et al*. [38] presented a hybrid learning framework that integrates a pretrained VGG19 model with a support vector machine classifier for the diagnosis of respiratory diseases using chest X-rays, verifying its diagnostic performance. Furthermore, Yi *et al*. [39] proposed a comprehensive lung disease classification model using data preprocessing, feature extraction, and classification modules. This model integrates ResNet, channel attention mechanisms, and dual Blaze-Block components within the RED-CNN framework, thereby enhancing feature extraction capabilities. Overall, these studies emphasize that architectural refinements, the incorporation of attention mechanisms, and advancements in classification methodologies greatly contribute to the accuracy improvement of pneumonia detection.

#### 2.2.2 Lung Cancer Detection and Diagnosis

According to statistical data provided by the World Health Organization, lung cancer causes approximately 7.6 million deaths annually, ranking it among the most lethal forms of cancer. In recent years, deep learning techniques using CT imaging have been as a pivotal approach in the field of computer-aided diagnosis. Current research mainly focuses on the detection of pulmonary nodules, differentiation between benign and malignant lesions, and the prediction of pathological subtypes. Jacobs *et al*. conducted an evaluation of multiple deep learning algorithms for lung cancer detection based on CT scans, evaluating leading models from the 2017 Kaggle Data Science Competition, such as grt123, Julian de Wit, Daniel Hammack, and Aidence [16]. Ozdemir *et al*. introduced a lung cancer diagnostic system using a three-dimensional CNN based on the LUNA16 and Kaggle datasets, demonstrating high efficiency in both nodule detection and malignant tumor classification [17]. Wang *et al*. proposed a deep CNN using an attention mechanism, which does not require pre-labeling nodule locations and can effectively integrate clinical demographic data [35]. Furthermore, Rajasekar *et al*. developed a deep learning framework that combines CT imaging with histopathological data, enhancing output performance relative to established architectures such as CNN, CNN GD, VGG16, VGG19, Inception V3, and ResNet50 [40]. Asuntha and Srinivasan presented a deep learning approach using CT images and feature extraction methods, including HOG, wavelet features, LBP, SIFT, and Zernike moments to classify various stages of lung cancer [13]. Additionally, Heidari *et al*. developed a deep learning model using blockchain-supported federated learning. The results were evaluated on CT datasets such as TCIA, Kaggle, and LUNA16, achieving a detection accuracy of 99.69% [41].

Ensemble learning techniques have been widely used in lung cancer classification. Quasar *et al*. introduced a lung cancer classification framework that considers BEiT, DenseNet, and Sequential CNN architectures [6]. This approach uses ensemble strategies including logical AND, OR operations, weighted box fusion, and boosting to enhance classification accuracy on CT scan images. In a similar vein, Gautam *et al*. developed an ensemble framework comprising three convolutional neural network models, namely ResNet152, DenseNet169, and EfficientNet [21]. Their method assigns weights to each model’s contribution based on performance metrics such as the receiver operating characteristic (ROC) curve, area under the curve (AUC), and F1 score, thereby achieving good lung cancer classification results on CT datasets. Trajanovski *et al*. proposed a comparable ensemble learning approach involving a two-stage deep learning model on a large-scale CT image dataset. This model facilitates the localization of lung nodules, evaluates the malignancy risk of adjacent tissues, and improves the overall detection accuracy of lung diseases [9]. Furthermore, Sultana *et al*. presented an ensemble method that combines features extracted from MobileNetV2, VGG19, and ResNet50 architectures, using weighted fusion techniques to enhance the detection and classification accuracy of lung diseases [20].

#### 2.2.3 Multiple Lung Disease Detection and Diagnosis

With the growing accessibility of CT imaging datasets and fast advancements in computational capabilities, there has been a surge in the development of deep learning approaches aimed at the simultaneous diagnosis of multiple pulmonary diseases. Oh *et al*. introduced an OView-AI system, which employs the EfficientNet-B7 architecture to classify pneumonia, pneumothorax, tuberculosis, and lung cancer from chest X-ray images, attaining an average test accuracy of 97.42% [14]. This system was verified on a dataset of 910 clinical cases, emphasizing its potential for clinical translation. Ibrahim *et al*. proposed a multi-classification framework that integrates chest X-ray and CT imaging modalities and evaluates several architectures, including VGG19-CNN and ResNet152V2, for the detection of COVID-19, pneumonia, and lung cancer [42]. Their findings indicate that the hybrid VGG19-CNN model has good prediction performance. In a similar vein, Rehman *et al*. developed three deep learning models based on X-ray images for the diagnosis of lung cancer, pneumonia, and COVID-19, showing that a CNN-LSTM hybrid model outperformed both standalone CNN and InceptionV3-LSTM architectures [43]. Additionally, Ashwini *et al*. presented a dual-model deep learning framework using grid search for hyperparameter optimization and successfully achieved multi-class classification results such as COVID-19, lung shadows, lung cancer, tuberculosis, and pneumonia [44].

### 2.3 Motivation and Objectives of the Proposed Ensemble Learning Method

As mentioned before, optimizing critical parameters of ensemble learning frameworks remains a methodological challenge in current research, especially in the field of medical imaging, where systematic methodologies addressing this issue are usually deficient. Though ensemble techniques have been widely applied to tasks such as pneumonia classification, lung cancer detection, and multi-disease diagnosis, few studies have deeply developed the adjustment of key hyperparameters (especially weight coefficients and decision thresholds) during the fusion process. Current approaches mainly rely on empirical parameter settings or exhaustive grid search methods, lacking an integrated framework that effectively balances simplicity, interpretability, and computational efficiency.

To address the limitations previously identified, we introduce a lightweight model fusion strategy based on weighted probability. This fusion framework enables the concurrent optimization of weight coefficients and decision thresholds, thereby simplifying the model fusion process while improving performance in practical applications. Notably, the proposed lightweight fusion approach does not require retraining any base models during the weighted fusion procedure. Its effectiveness has been demonstrated in the clinical differentiation between lung cancer and pneumonia. A comprehensive evaluation of nine neural network architectures, including established models such as VGG and the ResNet family, revealed that a ResNet variant achieved good performance. Distinct from conventional fusion techniques, such as stacking or majority voting, our method employs pairwise combinations of ResNet models, computes a weighted sum of their probability outputs using optimized weights, and determines the final classification based on a predetermined threshold. This approach effectively utilizes the complementary strengths of diverse architectures while preserving computational efficiency. Overall, this study presents a lightweight and robust fusion framework applied to lung disease diagnosis, thereby advancing clinical practice and methodologies in medical image analysis.

## 3 METHODS

### 3.1 Baseline Deep Learning Methods

#### 3.1.1 VGGNet Models

VGGNet was initially proposed by adopting a deep CNN architecture characterized by stacking multiple small 3×3 convolutional kernels and 2×2 max-pooling layers. Its convolutional block design can be generally formulated as Eq. (**1**):


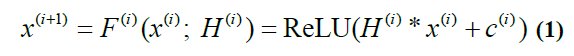


where *F*^(^*^i^*^)^ is a representing function. *x*^(^*^i^*^)^ represents its input feature map at layer *i*. *H*^(^*^i^*^)^ denotes a combination of 3×3 filters. * gives the convolution operation. *c*^(^*^i^*^)^ is a bias term. In this way, its deep sequential representation can be written as Eq. (**2**):


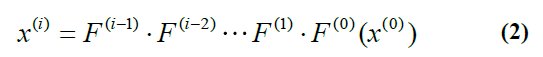


This modular design enhances network depth and representational capacity. Stacking two 3×3 convolutional layers can obtain an equivalent receptive field to a single 5×5 layer. The total parameter number of a convolutional layer can be calculated as Eq. (**3**):


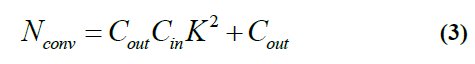


where *C_out_* and *C_in_* denote the parameter numbers of input and output channels, respectively. *K* represents the kernel size. Well-known variants, VGG16 and VGG19, have 13 and 16 convolutional layers, respectively, each followed by three fully connected layers.

Particularly, the output softmax function is written as Eq. (**4**):


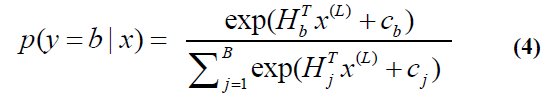


where *B* is the number of classes. *H_b_^T^* and *H_j_^T^* denote the weights for class *b* and class *j*, respectively. *c_b_* and *c_j_* represent the bias terms for class *b* and class *j*, respectively.

Due to its streamlined architecture and robust feature extraction capabilities, VGGNet has become a widely used model in the field of computer vision. Nonetheless, its extensive computational resource demands produce limitations for practical implementation.

#### 3.1.2 ResNet Models

ResNet was initially proposed to address the degradation problem encountered in deep neural networks as depth increases by introducing a deep residual learning framework. This architecture uses shortcut connections that bypass one or more layers, enabling direct information flow and expressing the output as *H*(*x*) = *F*(*x*) + *x*, where *F*(*x*) denotes the residual function. The fundamental residual block is mathematically formulated as Eq. (**5**):


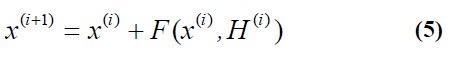


Through a recursive process, an input feature map at layer *L* can be written as Eq. (**6**):


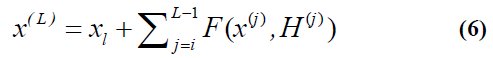


For backward propagation, given the loss function *ε*, its gradient with respect to the input *x*^(^*^i^*^)^ can be derived by using the following chain Eq. (**7**):


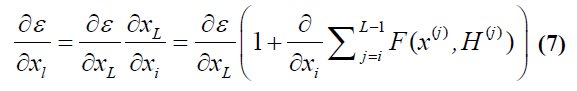


The total parameters of a standard residual block are approximated and calculated by Eq. (**8**):


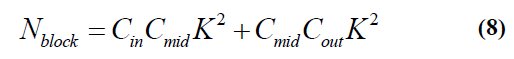


where *C_mid_* denotes the parameter number of the middle channel.

This design mitigates performance degradation by preserving identity mappings, thereby facilitating the addition of layers to improve representational ability without affecting training efficacy. ResNet has been widely used in various tasks, including image classification, object detection, and semantic segmentation, playing an important role in deep learning frameworks. However, its numerous depths and parameters require huge computational resources, which may increase the risk of overfitting in scenarios of limited data or less complex tasks. Accordingly, this study proposes a lightweight ensemble learning model fusion method based on post-hoc weighted averaging of predicted probabilities and aims to conduct an evaluation of multiple deep learning architectures for the diagnosis of pulmonary diseases. Specifically, we evaluate all models among the VGG family (VGG11, VGG13, VGG16, VGG19) and the ResNet family (ResNet34, ResNet50, ResNeXt50, ResNet101, ResNeXt101). Through comparative analysis of their classification performance, we determine the most effective models, which will subsequently serve as the theoretical foundation for establishing ensemble deep learning frameworks.

### 3.2 Ensemble Learning Strategy

All baseline models were initialized with pretrained weights and fine-tuned on our dataset. The optimizer was used with an initial learning rate of 0.001. The batch size was set to 32, and the models were trained for 50 epochs. Next, we will present an evaluation of the performance enhancements achieved by the selected ResNet-based models. Hence, we developed a lightweight ensemble deep learning fusion approach that combines two ResNet-based models. This ensemble configuration enhances robustness by enabling one model to mitigate the misclassifications of the other, thereby producing more accurate prediction performance. Its implementation details are illustrated in Fig. (**1**). The proposed lightweight ensemble fusion procedure is summarized in the following Algorithm.

**Algorithm: Lightweight Ensemble Learning via Weighted Probability Fusion:** 

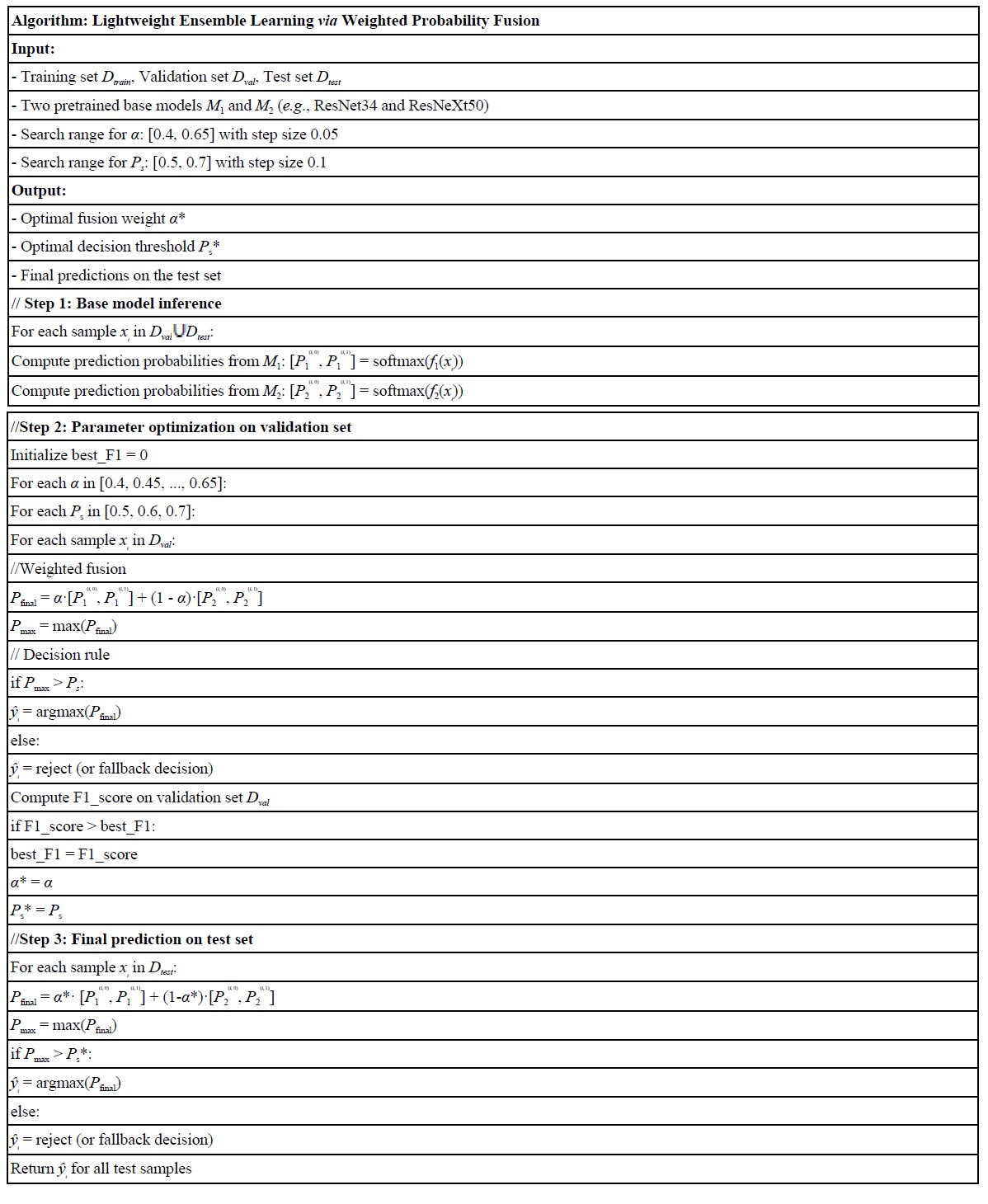

**Step 1** involves the extraction of probability vectors. For each input CT image, the initial prediction probabilities are obtained by using two pretrained ResNet models, such as ResNet34 and ResNet50. The resulting probability vectors derived from Net1 and Net2 are formally defined as Eqs. (**9** and **10**):


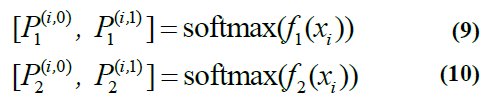


where subscripts 1 and 2 correspond to Net1 and Net2, respectively. The functions *f*_1_(*x_i_*) and *f*_2_(*x_i_*) represent the logits generated by these networks for sample *x_i_*. A superscript of (*i*, 0) denotes a pneumonia prediction for sample *x_i_*, while (*i*, 1) indicates a lung cancer prediction.

The softmax function converts a logit vector **z** = (*z*^(0)^, *z*^(1)^) into a probability distribution

***P*** = (*P*^(0)^, *P*^(1)^), and is defined for each class *c* as Eq. (**11**):


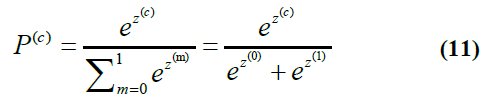


Thus, the probability vectors for Net1 and Net2 are formally computed as Eqs. (**12** and **13**):


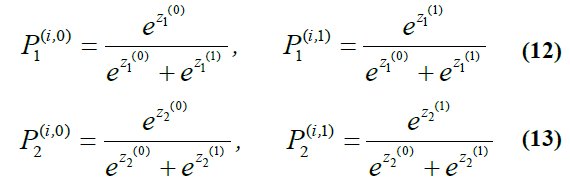


where *P_k_*^(^*^i^*^,0)^ and *P_k_*^(^*^i^*^,1)^ denotes the predicted probabilities for pneumonia and lung cancer, respectively, from model *k* for sample *x_i_*.

**Step 2** involves the weighted integration of the probability vectors. The resultant probability vector is derived as a weighted combination of the prediction probabilities generated by two models:


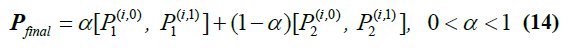


where the weighting coefficient *α* determines the relative influence of each model in the combined prediction, as shown in Eq. (**14**). This parameter should be optimized through validation data to improve diagnostic performance. An *α* value of 0.5 indicates equal weighting between the models, whereas values exceeding or falling below 0.5 indicate a higher degree of confidence in one model compared to the other.

The weighting coefficient *α* can be optimized by minimizing the cross-entropy loss over a validation set in Eq. (**15**):


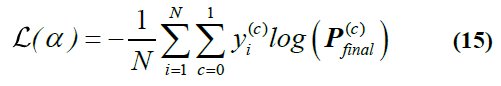


Where *y_i_*^(c)^ is the ground truth label in one-hot form. N is the number of samples in the validation set.

**Step 3** involves the application of a decision rule based on a predetermined threshold *P_s_*. The ultimate prediction is established by evaluating whether the maximum fused probability exceeds this threshold. Specifically, the maximum fused probability *P*^(^*^i^*^)^_max_ and its associated predicted category *ŷ_i_* are defined as following Eqs. (**16** and **17**):


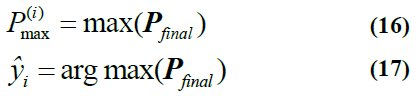


A prediction is considered reliable when the maximum probability *P*^(^*^i^*^)^_max_ surpasses the predetermined threshold *P_s_*, resulting in Eq. (**18**):


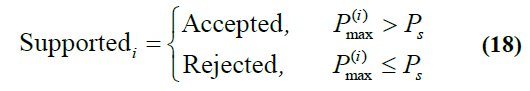


where the threshold *P_s_* can be optimized and adjusted according to actual clinical needs.

The proposed lightweight ensemble learning approach is characterized by its simplicity, interpretability, and parameterized framework. Compared to Bagging and Boosting, it leaves the training process unchanged. Compared to Stacking, it does not adopt a complex meta-model. Their main features are listed in Table **1**.

### 3.3 Dataset Description

This study used CT images obtained from more than 70 patients who were clinically diagnosed with either lung cancer or pneumonia. As shown in Fig. (**2**), the dataset was collected from the confirmed medical imaging cases maintained by the Department of Respiratory and Critical Care Medicine at Union Hospital, Tongji Medical College, Huazhong University of Science and Technology, China. All CT images were acquired using standard clinical multidetector CT scanners with routine scanning protocols. According to the details given in Table **2**, the complete dataset contains 859 images, including 503 images for training (including 238 cases of lung cancer and 265 cases of pneumonia), 176 images for validation (88 cases each of lung cancer and pneumonia), and 180 images for testing (90 cases each of lung cancer and pneumonia). The main objective of this dataset is to facilitate the clinical differentiation between lung cancer and pneumonia based on CT images. Selected cases are CT scans from patients with a clinical diagnosis of either lung cancer or pneumonia. Cases presenting with both conditions simultaneously were excluded from the dataset. Additionally, cases with patient gender or the stage of lung cancer were not considered in this dataset.

## 4 RESULTS AND DISCUSSIONS

All experiments were conducted using Python 3.7 with PyTorch 2.7.0 as the deep learning framework. The training and inference processes were performed on a workstation equipped with an Intel Core i5-10300H CPU (2.5 GHz) and 12 GB of RAM. CUDA was not utilized, as all computations were executed on the CPU. For reproducibility, the random seed was fixed to 42 across all experiments. The baseline deep learning models, including the VGG series (VGG11, VGG13, VGG16, VGG19) and the ResNet series (ResNet34, ResNet50, ResNeXt50, ResNet101, ResNeXt101), were implemented using standard architectures provided by the torchvision library.

### 4.1 Model Benchmarking

Table **3** gives the comparative performance of individual models in the classification task of lung cancer and pneumonia using CT images. The ResNet architecture always outperformed the VGG model. Specifically, four ResNet variants, namely ResNet34, ResNeXt50, ResNet50, and ResNeXt101, obtained accuracy levels over 0.91. Among them, ResNet34 produced the highest prediction performance, achieving an accuracy of 0.95, precision of 0.9355, recall of 0.9667, F1 score of 0.9504, and an AUC of 0.9924. However, the VGG models underperformed in this classification scenario, with the best-performing VGG13 model having an accuracy of 0.7333 and recall of 0.8333. This disparity in performance may be caused by the VGG model’s reliance on input images with a resolution of 224 × 224 × 3, which may be insufficient for capturing key features for accurate differentiation between these two diseases. In summary, these results verify the enhanced effectiveness of ResNet-based models in the classification of lung cancer and pneumonia from CT scanning images.

The residual learning framework adopted by ResNet efficiently mitigated the vanishing gradient problem commonly encountered in deep neural networks, thereby enabling more stable training of deeper architectures. For the CT-based classification task of lung cancer and pneumonia, deep networks are especially helpful because the two diseases show different yet subtle imaging features. Pneumonia often appears as patchy consolidations or ground-glass opacities spread across the lungs, while lung cancer usually shows up as a single nodule with irregular edges or spiculation. Telling them apart requires the model to pick up on both fine local details and the bigger picture of how the lesions are distributed. When it comes to the CT-based classification task of lung cancer and pneumonia, the extraction of subtle lesion characteristics requires the implementation of such deep networks. Residual connections facilitate the maintenance of salient lesion information among multiple layers by allowing low-level texture features to be preserved through identity mappings, thereby helping the model in discerning key imaging features for diagnosis while preventing the loss of critical diagnostic cues that may be diluted in plain networks like VGG. Empirical results verified the superiority of the ResNet architecture in this task. For example, ResNet34 obtained higher accuracy, recall, and F1 scores, whereas ResNeXt50 had superior precision. This complementary performance profile motivated the adoption of a lightweight ensemble learning approach, where multiple high-performing ResNet variants were integrated to yield classification results that outperformed those of any individual model.

### 4.2 Ensemble Model Performance Analysis

Tables **4** and **5** provide the prediction performance results of various ensemble model configurations, showing that the weighted prediction probability fusion approach enhanced classification accuracy for lung cancer and pneumonia detection. All ensemble techniques outperformed the individual models reported in Table **3** across multiple evaluation metrics such as accuracy, precision, recall, F1 score, and AUC. Furthermore, the ensemble consisting of ResNet34 and ResNeXt50, with parameters *α* = 0.6 and *P_s_* = 0.5, attained the highest accuracy (0.9667), recall (0.9888), and F1 score (0.9674). Figure **3** presents the confusion matrix, PR curve, and ROC curve, further demonstrating that the ensemble model outperformed the ResNet34 model across all evaluated metrics. The observed performance enhancement of the ensemble model can be attributed to the complementary capabilities of ensemble models. When one model misclassifies a sample, the other model may provide a correct diagnosis. The weighted probability fusion strategy effectively mitigates classification errors by using these complementary strengths. For example, a single ResNet34 model yields 6 false positives (FPs) and 3 false negatives (FNs), whereas a single ResNeXt50 model produces 6 FPs and 4 FNs. Integration of these models reduces these errors to 5 FPs and 1 FN, combining the fusion of ResNeXt50’s false positive tendencies with ResNet34’s false negative advantages. Considering the serious health risks of misdiagnosing lung cancer or pneumonia, this error-correcting capacity shows important clinical significance.

The weighting coefficients *α* and the decision threshold *P_s_* play an important role in determining the ensemble model performance. As shown in Table **5**, increasing *α* from 0.4 to 0.6 provided enhancements in accuracy, precision, recall, and F1 score across all ensemble configurations. These findings suggest that assigning larger weights to more robust individual models could enhance the ensemble classification outcomes. Importantly, for the ensemble model comprising ResNet34 and ResNeXt50, diagnostic accuracy increased from 0.9500 at *α* = 0.4 to 0.9667 at *α* = 0.6, showing an ensemble accuracy improvement of 1.67%. However, further elevation of *α* to 0.65 did not produce additional gains, demonstrating that heavy reliance on individual models may weaken the benefits of ensemble approaches. Additionally, increasing the threshold *P_s_* from 0.5 to 0.7 resulted in performance degradation of all ensemble models, mainly due to a reduction in the number of samples that benefit from error correction. The weight and threshold parameters were optimized as *α* = 0.6 and *P_s_* = 0.5 The optimized parameters can effectively balance the influence of stronger models and preserve the complementary corrections of other models, which prevents the excessive exclusion of cases requiring re-assessment. This optimized ensemble learning framework is expected to enhance the diagnostic accuracy for lung cancer and pneumonia, thereby providing radiologists with a more dependable decision support tool.

Several limitations should be acknowledged. First, our dataset was collected from a single institution and contains 859 CT images, which is relatively modest in size. Whether the proposed method generalizes to larger, multi-institutional datasets remains to be tested. Second, this study focused only on differentiating pneumonia and lung cancer. The applicability of our method to other pulmonary diseases with overlapping imaging features, such as tuberculosis or interstitial lung disease, has not been explored.

## CONCLUSION

This paper presents a lightweight ensemble learning model fusion method based on post-hoc weighted averaging of predicted probabilities. The proposed lightweight ensemble learning approach simultaneously optimizes two pivotal parameters: the weight coefficient and the probability decision threshold. This dual-parameter optimization outperformed conventional model averaging techniques by effectively utilizing the complementary characteristics of individual models, which thereby contributed to a reduction in prediction errors. Importantly, the proposed lightweight ensemble learning framework does not require retraining the selected base models during the whole ensemble process. Comprehensive evaluations were conducted on medical image classification tasks, specifically involving pneumonia and lung cancer datasets. The experimental results demonstrate that the proposed method enhanced critical performance metrics such as accuracy, precision, recall, and F1-score for various model combinations, with the best performance achieved by the fusion of ResNet34 and ResNeXt50 (*α* = 0.6, *P_s_* = 0.5), yielding an accuracy of 96.67%, precision of 94.68%, recall of 98.88%, and F1-score of 96.74%. These results validated the effectiveness of the proposed lightweight ensemble learning framework.

Future work can focus on extending the generality of the proposed lightweight ensemble learning framework. This includes exploring its implementations in more complex computer vision tasks, such as semantic segmentation and object detection, where probabilistic fusion may be applied at the pixel or regional level. Additionally, investigations into adaptive optimization of key parameters will be pursued to enable self-calibration mechanisms capable of accommodating dynamic data streams and distributional shifts. In particular, we plan to explore adaptive fusion strategies that can dynamically adjust the weighting coefficient and decision threshold based on input image characteristics or model confidence, which would improve the generalizability of the proposed method across different diseases, imaging centers, patient populations, and imaging modalities.

## Figures and Tables

**Fig. (1):**
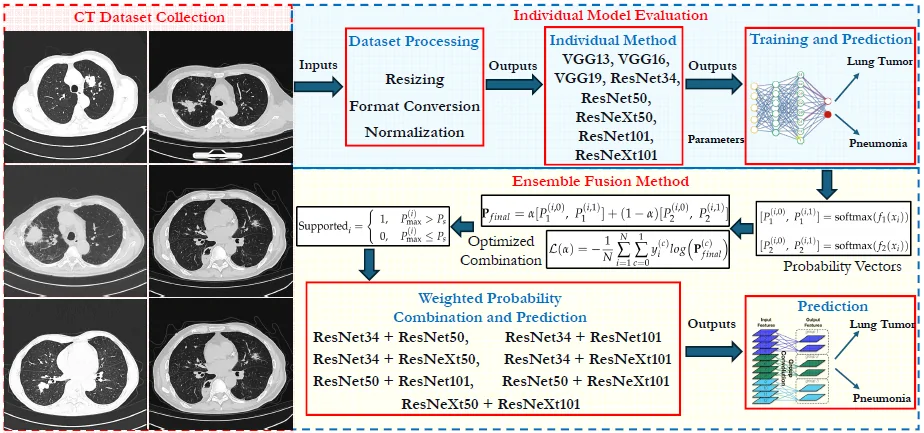
Block diagram of the proposed lightweight ensemble deep learning fusion method.

**Fig. (2):**
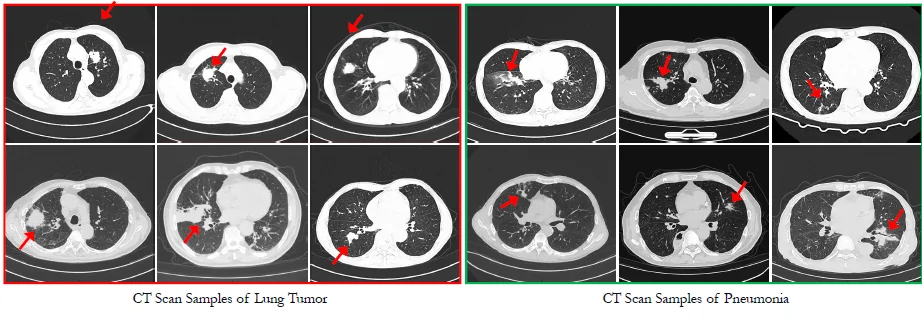
CT scan samples of lung cancer and pneumonia.

**Fig. (3):**
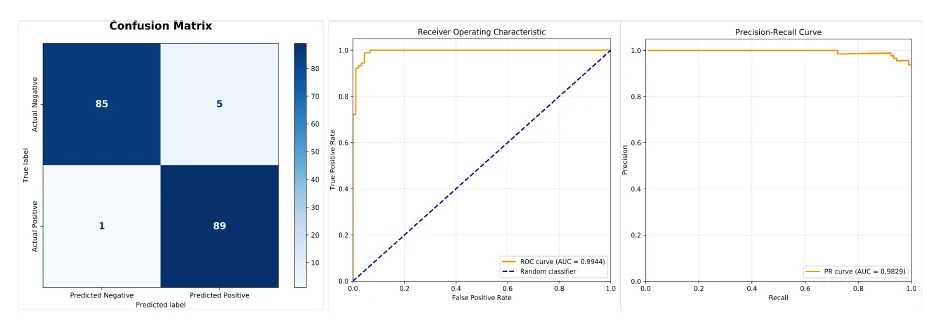
Confusion matrix, ROC Curve, and PR Curve results for the combination of ResNet34 and ResNeXt50 with *α* = 0.6 and *P_s_* = 0.5.

**Table 1:** Ensemble learning comparison of the proposed method with bagging, boosting, and stacking.

** Features **	** Proposed **	** Bagging **	** Boosting **	** Stacking **
Main idea	Linearly weights the prediction probabilities of pretrained models.	Constructs multiple models *via* bootstrap sampling, combining them through equal voting.	Trains models sequentially, with each new model focusing on correcting its predecessors.	Uses base model predictions as new features to train a meta-model for optimal combination.
Training process	Base models are trained independently; fusion occurs only during inference *via* weighted averaging.	Base learners are trained in parallel.	Base learners are trained sequentially.	All base models are trained first, followed by the meta-model.
Interpretability	The fusion logic is a clear, intuitive linear formula.	Can be interpreted as a “majority vote”.	The complex dependencies between models make it a “black box”.	The meta-learner itself is a black box, and difficult to explain the logic behind the combination.
Base models	Aims to combine complementary, pretrained models.	Forces the same weak model to learn different aspects of the data through data sampling.	Adjusts data weights to make subsequent models focus on difficult samples.	Allows the meta-model to discover relationships between the base models

**Table 2:** Datasets collection.

**Items**	**Lung Cancer**	**Pneumonia**	**Total**
Training sets	238	265	503
Validation sets	88	88	176
Testing sets	90	90	180

**Table 3:** Individual model comparison results.

**Model**	**TN**	**FP**	**FN**	**TP**	**Accuracy**	**Precision**	**Recall**	**F1 Score**	**AUC**
VGG11	16	74	10	80	0.5333	0.5195	0.8889	0.6557	0.5775
VGG13	51	39	15	75	0.7	0.6579	0.8333	0.7353	0.7537
VGG16	15	75	7	83	0.5444	0.5253	0.9222	0.6694	05044
VGG19	86	4	79	11	0.5389	0.7333	0.1222	0.2095	0.5750
**ResNet34**	**84**	**6**	**3**	**87**	**0.95**	**0.9355**	**0.9667**	**0.9504**	**0.9924**
**ResNet50**	**78**	**12**	**4**	**86**	**0.9111**	**0.8775**	**0.9555**	**0.9149**	**0.9560**
**ResNeXt50**	**84**	**6**	**4**	**86**	**0.9444**	**0.9348**	**0.9556**	**0.9450**	**0.9894**
**ResNet101**	**77**	**13**	**7**	**8**3	**0.8889**	**0.8646**	**0.9222**	**0.8925**	**0.9664**
**ResNeXt101**	**84**	**6**	**10**	**80**	**0.9111**	**0.9302**	**0.8889**	**0.9091**	**0.9827**

**Table 4:** Ensemble model results with weighting coefficient *α* = 0.6 and preset threshold *P_s_* = 0.5.

**Model**	**TN**	**FP**	**FN**	**TP**	**Accuracy**	**Precision**	**Recall**	**F1 Score**	**AUC**
**ResNet34 + ResNet50**	**84**	**6**	**1**	**89**	**0.9611**	**0.9368**	**0.9888**	**0.9621**	**0.9926**
ResNet34 + ResNet101	84	6	2	88	0.9555	0.9361	0.9777	0.9565	0.9938
**ResNet34 + ResNeXt50**	**85**	**5**	**1**	**89**	**0.9667**	**0.9468**	**0.9888**	**0.9674**	**0.9941**
**ResNet34 + ResNeXt101**	**86**	**4**	**3**	**87**	**0.9611**	**0.9560**	**0.9666**	**0.9613**	**0.9946**
ResNet50 + ResNet101	82	8	4	86	0.9333	0.9148	0.9555	0.9347	0.9849
ResNet50 + ResNeXt101	86	4	4	86	0.9555	0.9555	0.9555	0.9555	0.9844
**ResNeXt50 + ResNeXt101**	**87**	**3**	**4**	**86**	**0.9611**	**0.9662**	**0.9555**	**0.9911**	**0.9912**

**Table 5:** Ensemble model comparison results with different weighting coefficient *α* and threshold *P_s_*.

**Model**	***α***	***Ps***	**Accuracy**	**Precision**	**Recall**	**F1 Score**	**AUC**
ResNet34 + ResNet50	0.4	0.5	0.9333	0.9148	0.9555	0.9347	0.9900
	0.5	0.5	0.9555	0.9361	0.9777	0.9565	0.9923
	**0.6**	**0.5**	**0.9611**	**0.9368**	**0.9888**	**0.9621**	**0.9926**
	**0.65**	**0.5**	**0.9611**	**0.9368**	**0.9888**	**0.9621**	**0.9930**
	0.6	0.6	0.9388	0.9157	0.9666	0.9405	0.9926
	0.6	0.7	0.8888	0.8571	0.9333	0.8936	0.9926
ResNet34 + ResNet101	0.4	0.5	0.9111	0.8854	0.9444	0.9139	0.9901
	0.5	0.5	0.9222	0.8877	0.9666	0.9255	0.9920
	**0.6**	**0.5**	**0.9555**	**0.9361**	**0.9777**	**0.9565**	**0.9938**
	**0.65**	**0.5**	**0.9555**	**0.9361**	**0.9777**	**0.9565**	**0.9941**
	0.6	0.6	0.9111	0.8775	0.9555	0.9148	0.9938
	0.6	0.7	0.8833	0.8484	0.9333	0.8888	0.9938
**ResNet34 + ResNeXt50**	0.4	0.5	0.9500	0.9450	0.9555	0.9502	0.9938
	0.5	0.5	0.9555	0.9361	0.9777	0.9565	0.9937
	**0.6**	**0.5**	**0.9667**	**0.9468**	**0.9888**	**0.9674**	**0.9941**
	**0.65**	**0.5**	**0.9667**	**0.9468**	**0.9888**	**0.9674**	**0.9941**
	0.6	0.6	0.9555	0.9361	0.9777	0.9565	0.9941
	0.6	0.7	0.9222	0.9042	0.9444	0.9239	0.9940
ResNet34 + ResNeXt101	0.4	0.5	0.9388	0.9540	0.9222	0.9378	0.9922
	0.5	0.5	0.9500	0.9450	0.9555	0.9502	0.9941
	**0.6**	**0.5**	**0.9611**	**0.9560**	**0.9666**	**0.9613**	**0.9946**
	0.65	0.5	0.9555	0.9456	0.9666	0.9560	0.9948
	0.6	0.6	0.9333	0.9148	0.9555	0.9347	0.9946
	0.6	0.7	0.8944	0.8817	0.9111	0.8961	0.9946
ResNet50 + ResNet101	0.4	0.5	0.9222	0.9042	0.9444	0.9239	0.9828
	0.5	0.5	0.9222	0.9042	0.9444	0.9239	0.9222
	**0.6**	**0.5**	**0.9333**	**0.9148**	**0.9555**	**0.9347**	**0.9849**
	0.65	0.5	0.9277	0.9052	0.9555	0.9297	0.9850
	0.6	0.6	0.8833	0.8484	0.9333	0.8888	0.9849
	0.6	0.7	0.8666	0.8235	0.9333	0.8749	0.9849
ResNet50 + ResNeXt101	0.4	0.5	0.9444	0.9444	0.9444	0.9444	0.9834
	0.5	0.5	0.9444	0.9444	0.9444	0.9444	0.9839
	**0.6**	**0.5**	**0.9555**	**0.9555**	**0.9555**	**0.9555**	**0.9843**
	0.65	0.5	0.9444	0.9347	0.9555	0.9450	0.9841
	0.6	0.6	0.9166	0.8947	0.9444	0.9189	0.9843
	0.6	0.7	0.8833	0.8556	0.9222	0.8877	0.9843
ResNeXt50 + ResNeXt101	0.4	0.5	0.9444	0.9651	0.9222	0.9431	0.9904
	0.5	0.5	0.9555	0.9767	0.9333	0.9545	0.9912
	**0.6**	**0.5**	**0.9611**	**0.9662**	**0.9555**	**0.9608**	**0.9911**
	**0.65**	**0.5**	**0.9611**	**0.9662**	**0.9555**	**0.9608**	**0.9908**
	0.6	0.6	0.9500	0.9655	0.9333	0.9491	0.9911
	0.6	0.7	0.9111	0.9021	0.9222	0.9120	0.9911

## Data Availability

The data and supportive information are available within the article.

## LIST OF ABBREVIATIONS

CT: Computed tomography
VGG: Visual Geometry Group
ResNet: Residual Network
DNNs: Deep neural networks
CNNs: Convolutional neural networks
AI: Artificial intelligence
ViT: Vision transformer
ROC: Receiver operating characteristic
AUC: Area under the curve
FPs: False positives
FNs: False negatives
